# Single-cell counting reveals a modest, nutrient-dependent growth advantage of early *Pseudomonas fluorescens* biofilms over planktonic cells

**DOI:** 10.1016/j.bioflm.2026.100408

**Published:** 2026-10-29

**Authors:** Jacobus Brink, Dishon Wayne Hiebner, Eoin Casey

**Affiliations:** UCD School of Chemical and Bioprocess Engineering, Dublin, Ireland

**Keywords:** *Pseudomonas fluorescens*, Biofilm, Planktonic growth, Specific growth rate, Carbon source, Biofilm architecture

## Abstract

Biofilm and planktonic cells differ in many physiological traits, yet whether attached cells grow faster than planktonic cells under the same conditions has rarely been measured with comparable methods. Here, we used confocal microscopy and deep-learning-based segmentation to count individual cells in early *Pseudomonas fluorescens* WCS365 biofilms. We then compared their specific growth rates (μ) with those of planktonic cultures in the same M9 medium, with glucose, glycerol, or succinate supplied at equal carbon concentration, with and without Casamino Acids (CAA). With CAA, attached cells grew on average 15% faster than planktonic cells (95% CI 7–24%), and this difference did not depend significantly on the carbon source. Growth rates followed the same order in both lifestyles (glucose > glycerol > succinate). Removing CAA at matched total carbon reduced growth rates by 42–66% where growth was detected, and its effect on the biofilm advantage differed between carbon sources. Specifically, the advantage disappeared in glucose, whereas in glycerol, attached cells grew while planktonic cultures showed no measurable growth within 8 h. Carbon source also shaped biofilm architecture: at 8 h, biofilm volume aligned with growth rates, but glycerol biofilms were the tallest and cells in succinate biofilms were spaced further apart. Together, these findings show that surface-attached *P. fluorescens* grows on average 15% faster than planktonic cells in amino-acid-supplemented media, that this advantage depends on the nutritional context rather than on attachment alone, and that carbon source shapes biofilm structure in ways not solely explained by growth rate.

## Highlights

• 3D confocal imaging and deep learning segmentation measured biofilm cell growth rates.
• Attached *P. fluorescens* grew 15% faster than planktonic cells with CAA.
• Surface growth advantage depends on nutrient context, not attachment alone.
• Carbon source shapes early biofilm architecture beyond cell growth rate.

## 1 Introduction

Microbial biofilms are surface-attached communities embedded in a self-produced matrix of extracellular polymeric substances (EPS). Their resilience to environmental stressors and antimicrobial agents underlies persistent clinical infections, industrial biofouling, and survival in natural environments [1], [2]. Biofilm-associated and planktonic cells differ markedly in physiology and gene expression [3]. Whether these differences extend to growth rate has been debated for decades: early studies reported that attachment to a surface can either stimulate or inhibit bacterial activity [4]. This question remains poorly resolved, however, partly because the two lifestyles are rarely evaluated using comparable methods.

The nutrient environment is one of the strongest influences on biofilm formation. In *Pseudomonas fluorescens* WCS365, the ability to initiate a biofilm on an abiotic surface depends on the nutrients available, and different nutrients act through distinct, converging regulatory pathways [5]. In *P*. *fluorescens* Pf0-1, phosphate limitation lowers intracellular c-di-GMP levels and blocks secretion of the adhesin LapA to the cell surface, preventing biofilm formation [6]. In *P. aeruginosa*, whether quorum sensing is required for biofilm development depends on the carbon source [7]. Additionally, a sudden increase in carbon substrates such as succinate, glutamate or glucose triggers biofilm dispersal [8]. These studies mostly measured parameters such as initial attachment, total biofilm biomass, biofilm structure, dispersal, or the underlying signalling pathways. How specific nutrients influence the rate at which cells grow within a biofilm, and whether this differs from planktonic growth under the same conditions, have received much less attention.

Part of the reason for this gap in knowledge is methodological. Biofilm formation is most often quantified as total attached biomass, for example by crystal violet staining, or as biovolume and thickness acquired from confocal images [9], [10]. These measures include the matrix and depend on cell size, and endpoint assays such as crystal violet staining do not provide growth rates. Conversely, planktonic growth is usually monitored by optical density (OD) or colony counts, which report population biomass or viable cell number in suspension. Comparisons between the two lifestyles therefore often rely on different quantities measured with different methods. Counting individual cells within biofilms would provide a direct measure of biofilm population growth, but dense, three-dimensional biofilms have been difficult to segment into single cells. Recent advances in confocal microscopy and deep-learning-based segmentation now make it possible to count cells within early biofilms and to measure their growth rates [11], [12].

*Pseudomonas fluorescens* is a widespread colonist of soil, plant surfaces, and water and a well-established model for surface colonisation and early biofilm formation [5], [13]. In this study, we used single-cell confocal imaging and automated segmentation to measure the specific growth rate of surface-attached *P. fluorescens* WCS365 from cell counts and compared it with that of planktonic cultures grown in identical media. Planktonic growth was measured by OD, which was validated against viable cell counts. Casamino Acids (CAA), an acid hydrolysate of casein, are commonly added to minimal media in biofilm assays [14], [15], but they also supply amino acids as additional carbon and nitrogen sources, and individual amino acids can modulate surface-associated behaviour in *Pseudomonas* [16]. Growth was therefore compared in three carbon sources (glucose, glycerol, and succinate) supplied at equimolar carbon concentrations, with and without CAA supplementation, to test whether the relative growth of attached and planktonic cells depends on the nutritional context. Finally, biofilm architecture was characterised at the end of the 8 h assay to examine whether differences in growth were reflected in biofilm structure.

## 2 Methods

### 2.1 Materials

Glycerol (C_3_H_8_O_3_), Tryptone (vegetable), Yeast Extract, Glucose (C_6_H_12_O_6_), Sodium Succinate Dibasic Hexahydrate (C_4_H_4_Na2O_4_·6H_2_O) (hereafter Succinate), Manganese (II) Chloride Tetrahydrate (MnCl_2_·4H_2_O), M9 Minimal Salts (15 g/L KH_2_PO_4_, 2.5 g/L NaCl, 33.9 g/L Na_2_HPO_4_ and 5 g/L NH_4_Cl), and Agar were purchased from Merck, Ireland. Calcium Chloride Anhydrous (CaCl_2_), Magnesium Sulfate Heptahydrate (MgSO_4_·7H_2_O), Iron (III) Chloride Hexahydrate (FeCl_3_·6H_2_O), Zinc Sulfate Heptahydrate (ZnSO_4_·7H_2_O), Copper (II) Sulfate Pentahydrate (CuSO_4_·5H_2_O), Sodium Molybdenum Oxide Dihydrate (Na_2_MoO_4_·2H_2_O), Difco™ Casamino Acids, Phosphate-Buffered Saline (PBS), Paraformaldehyde (PFA), and the LIVE/DEAD™ BacLight™ Bacterial Viability Kit was purchased from Thermo Fisher Scientific (Illinois, USA). Sodium Chloride (NaCl) was purchased from Fluorochem, UK. All reagents were used as provided.

### 2.2 Bacterial strain and culture conditions

The bacterial strain used in this investigation was *P. fluorescens* WCS365. Bacterial cultures were stored at −80 °C in Milli-Q H_2_O supplemented with 25% volume per volume (v/v) glycerol. To cultivate, thawed aliquots were streaked onto LB agar plates (Lennox formulation: 10 g/L tryptone, 5 g/L yeast extract, 5 g/L NaCl, and 15 g/L agar) and were incubated at 30 °C for 24 h. A single colony of *P. fluorescens* was used to inoculate 50 mL of M9 medium in a 250 mL Erlenmeyer flask. The medium was prepared as follows: M9 Minimal Salts (11.28 g/L) were dissolved in distilled H_2_O and autoclaved at 121 °C for 15 min (min). The following components were added aseptically after autoclaving by filter sterilisation (0.22 μm): MgSO_4_, CaCl_2_, Casamino Acids, and a trace element solution, yielding final concentrations of MgSO_4_ (2 mM), CaCl_2_ (0.1 mM), Casamino Acids (0.2% weight per volume (w/v)), FeCl_3_·6H_2_O (100 μM), MnCl_2_·4H_2_O (10 μM), ZnSO_4_·7H_2_O (10 μM), CuSO_4_·5H_2_O (1 μM) and Na_2_MoO_4_·2H_2_O (1 μM).

One of three carbon sources was added, each normalised to an equivalent carbon concentration of 120 mM C: glucose (20 mM), glycerol (40 mM), or succinate (30 mM). As CAA also supplies carbon, media with CAA were not single-carbon-source media, and the three carbon sources are referred to as primary carbon sources. To assess the contribution of CAA, each carbon source was also tested in medium without CAA. In these conditions, the carbon source concentration was increased to 160 mM C (glucose 26.7 mM, glycerol 53.3 mM, and succinate 40 mM), so that total carbon was matched to the CAA-supplemented medium. The approximately 40 mM C contributed by 0.2% (w/v) CAA was estimated from the total nitrogen content on the product Certificate of Analysis (7.2% N by weight) and the carbon-to-nitrogen ratio of casein amino acids. For every condition, overnight precultures were grown in the same medium as the subsequent growth assay. Cultures were incubated overnight (14–16 h) at 30 °C with shaking at 200 revolutions per minute (rpm). Precultures with CAA reached an OD at 600 nm (OD_600_) of approximately 2.3–2.6, whereas precultures without CAA reached lower densities over the same incubation time.

### 2.3 Planktonic growth curve assay

Planktonic growth experiments were performed in 250 mL Erlenmeyer flasks containing 50 mL of M9 medium with the respective carbon source, with or without CAA. Overnight cultures were diluted in fresh medium to an initial OD_600_ of 0.01 and incubated at 30 °C with shaking at 200 rpm. OD_600_ was measured every 60 min over 8 h using an ultraviolet-visible (UV-vis) spectrophotometer (Libra S11, Biochrom). When OD_600_ approached 0.8, samples were diluted in fresh medium prior to measurement, and the recorded value was multiplied by the corresponding dilution factor to obtain the corrected OD_600_. Specific growth rates (μ, h^−1^) were determined by fitting a linear regression to the natural logarithm of corrected OD_600_ values during the exponential growth phase (see Estimation of Specific Growth Rates and Doubling Time). With CAA, corrected OD_600_ tracked viable cell number over the fitted window (see OD_600_ Linearity and Viable Counts). The experiment was performed in three independent biological replicates, each with three technical replicates (separate flasks).

### 2.4 OD_600_ linearity and viable counts

To test whether OD_600_ was proportional to cell number, one culture per carbon source (with CAA) was grown as described above to an OD_600_ of approximately 1.3–1.8 and serially diluted twofold in fresh medium (eight concentrations, three independent dilution series, three readings each). Proportionality was assessed from the slope of log(OD_600_) against log(relative concentration) over the OD_600_ range used for growth rate estimation (0.024–0.687), where a slope of 1 indicates a proportional response. To compare OD_600_ with viable cell number in growing cultures, three biological replicate cultures per carbon source (with CAA) were inoculated at an OD_600_ of 0.01 and sampled at 0 h and hourly from 2 to 6 h. At each time point, OD_600_ was measured and colony-forming units (CFU) were determined by drop plating [17], [18]: tenfold serial dilutions in 0.85% (w/v) NaCl were spotted in triplicate 20 μL drops on LB agar and incubated at 30 °C for 24 h. The change in CFU per OD_600_ unit between 2 and 6 h was tested by regressing ln(CFU/OD_600_) against time, with a separate intercept for each culture and a common slope. Specific growth rates were calculated from CFU and from OD_600_ over the same window as the main planktonic experiment (glucose and glycerol: 2–5 h; succinate: 2–6 h) and compared as paired differences for each culture.

### 2.5 Fixation and staining of surface-attached biofilms

Overnight cultures were diluted 1:5000 (2 × 10^−4^) in fresh M9 medium with the respective carbon source, with or without CAA. A volume of 150 μL of each diluted culture was inoculated into the wells of a 96-well μ-Plate with a #1.5H borosilicate glass coverslip bottom and untreated surface (μ-Plate 96 Well Round, ibidi GmbH, Cat. No. 89607). Plates were incubated statically at 30 °C for 10 min to allow initial surface attachment. Wells were then washed three times with 200 μL of the corresponding sterile M9 medium to remove non-adherent cells and replenished with 150 μL of fresh medium of the same composition. Plates were subsequently incubated statically at 30 °C for 8 h to allow biofilm development.

Biofilms were fixed with an equal volume (150 μL) of 8% (w/v) PFA in 2× PBS (final concentration of 4% (w/v) PFA in 1× PBS) at hourly intervals for 8 h, immediately following the attachment and rinsing steps. Wells containing fixative were sealed with a plate sealer (Thermo Fisher Scientific, USA) to prevent evaporation, and the plate was incubated overnight at 30 °C to complete fixation. Following fixation, each well was washed three times with 0.85% (w/v) NaCl. Once all colonies were fixed, they were stained with SYTO™ 9 (3.34 μM final concentration) for 15 min at room temperature (RT) in the dark. Unbound dye was removed by washing three times with 0.85% (w/v) NaCl. Wells were then filled with 0.85% (w/v) NaCl and prepared for microscopy. Measurements were performed on three biological replicates, with 10 colonies imaged per replicate and time point as technical replicates.

### 2.6 Confocal microscopy acquisition and processing

All confocal z-stack images were acquired using a confocal laser scanning microscope LSM 900 equipped with a Plan-Apochromat 40×/1.3 Oil DIC (UV) VIS-IR M27 objective (Zeiss, Germany). SYTO™ 9 was excited with the 488 nm laser line (0.02% laser power), and emission was detected from 400 to 650 nm. Image stacks consisted of 10–60 optical sections with a z-step interval of 0.31 μm. Images were acquired with a scan zoom of 1.5, bidirectional scanning, and a pixel time of 0.67 μs. Each image had a resolution of 1163 × 1163 pixels, corresponding to a field of view of ∼106.5 μm × 106.5 μm. The GaAsP-PMT detector was operated at a gain of 600 V, with a detector digital gain of 0.3–0.7, and images were recorded at 16-bit depth. Averaging was performed line-wise (2×), and images were acquired at a frame time of 2.12 s.

### 2.7 Image segmentation and quantitative analysis

Individual cells in 3D image stacks were segmented with a custom Python pipeline implementing StarDist [19] with Optimised Post-Processing (OPP) [12]. Prior to segmentation, image stacks were cropped to regions of interest containing the developing colony. For biofilms grown with CAA, stacks at 2 h were cropped to 100 × 100 pixels, 3 h to 200 × 200 pixels, 4 h to 400 × 400 pixels, and 5 h to 600 × 600 pixels; stacks at 6, 7, and 8 h were processed without cropping. Biofilms grown without CAA, which remained smaller, were cropped to smaller regions. A single channel was extracted and normalised to the 1st and 99.8th percentiles. Cells were segmented with a pre-trained StarDist3D model (model 7) at its optimised probability and non-maximum suppression thresholds (0.4537 and 0.3). Because the model was trained on images with a z:xy voxel ratio of 2.3, whereas the present images had a ratio of 3.4 (0.31 μm in z and 0.0916 μm in xy), stacks were resampled in z by a factor of 1.47 before segmentation. Label images were resampled back to the original size by nearest-neighbour interpolation. Volumes of up to 2 × 10^6^ voxels were processed as a single tile, and larger volumes in overlapping blocks to limit memory use. StarDist predictions were merged into cell masks with the naive_fusion function of the merge-stardist-masks package, with no full overlaps permitted per object, which reduced the merging of touching cells compared with the original setting of two. Label images were exported as 16-bit TIFF stacks for analysis in BiofilmQ. Biofilms grown with and without CAA were segmented with the same settings.

Biofilm images were further analysed using BiofilmQ v1.0.1 [11]. The StarDist label images were imported as pre-segmented cells (label image-based segmentation), with the SYTO™ 9 channel as the intensity channel. From the global summary output files generated by BiofilmQ, the number of cells per colony was extracted and all quantified parameters were exported as CSV files for downstream analysis and visualisation.

### 2.8 Segmentation validation

Segmentation accuracy was validated against blinded manual point annotation of 30 image stacks, covering all three carbon sources with and without CAA, time points from 2 to 8 h, and low- and high-density biofilms. Accuracy was further benchmarked against manual outlines of 77 cells in 8 h biofilms (Supplementary Information, Segmentation Validation). Annotation was performed in Fiji [20].

### 2.9 Estimation of specific growth rates and doubling time

The specific growth rate (μ) of planktonic populations was estimated from corrected OD_600_, and that of biofilm populations from the number of segmented cells per colony. For each biological replicate, technical replicates (three flasks or 10 colonies) were averaged to give one value per time point, and μ was estimated as the slope of a linear regression of the natural logarithm of this value against time over the exponential phase (Supplementary Figs. S1 and S2). The exponential phase was identified from the hour-to-hour specific growth rate, ln(y_t_/y_t−1_) (Supplementary Fig. S3). For planktonic cultures with CAA, the window included all consecutive hourly intervals in which this rate was at least 80% of its maximum, excluding the first 2 h, which included the lag after inoculation. This gave 2–5 h for glucose and glycerol and 2–6 h for succinate; in the following interval, the rate fell from 0.8 to 1.1 h^−1^ to 0.6–0.7 h^−1^. Hour-to-hour biofilm rates were more variable, so a common window of 3–7 h was used. The 2 h time point was excluded because colonies contained only 2–5 cells, meaning a change of a single cell altered the count by 20–50%. The 8 h time point was also excluded because, with CAA, growth slowed between 7 and 8 h in all conditions (0.3–0.6 h^−1^, compared with 0.9–1.2 h^−1^ over 3–7 h). Without CAA, growth did not slow within the 8 h assay in either lifestyle, so the same 3–7 h window was used for biofilm and planktonic cultures to compare both over the same period. With CAA, windows extending beyond these time points gave lower growth rates and systematic residuals (Supplementary Fig. S4), whereas without CAA, extending the window to 2–8 h changed μ by no more than 0.03 h^−1^ (Supplementary Table S1). The goodness of fit (R^2^) was calculated for each regression. Doubling times were calculated as t_d_ = ln(2)/μ from the mean growth rate of each condition.

### 2.10 Statistical analysis

Statistical analyses were performed in R v4.6.1 within Positron (v2026.09.1). Specific growth rates were analysed at the level of biological replicates (n = 3 per group). Because differences in growth rate were compared as relative (percentage) differences and their variability differed between groups, μ was log-transformed and analysed with generalised least squares models that allowed a separate variance for each group (nlme package v3.1-170) [21], with Satterthwaite degrees of freedom (emmeans package v2.0.4) [22]. For media with CAA, the model included lifestyle, carbon source, and their interaction. A second model, restricted to glucose and succinate because no planktonic data were recorded for glycerol without CAA, additionally included CAA and all interactions. Effects were tested with *F*-tests, and biofilm-versus-planktonic differences, changes in these differences on removal of CAA, and differences between carbon sources were estimated as ratios of geometric means and reported as percentage differences with 95% confidence intervals. Confidence intervals and p-values for each set of comparisons were Bonferroni-adjusted.

Biofilm architectural properties were compared between the carbon sources with CAA at the endpoint (8 h). Five metrics were assessed: biofilm height (μm), biofilm volume (μm^3^), biofilm base area (μm^2^), mean distance to nearest-neighbour (μm), and mean distance inter-cell spacing (μm). Because multiple colonies were imaged from a shared overnight culture and media batch within each of three independent experimental days (biological replicates), colony-level measurements were not treated as fully independent to avoid pseudoreplication. For each metric, a linear mixed-effects model was fitted with growth medium as a fixed effect and biological replicate as a random intercept (lme4 package v2.0-6) [23]. Pairwise differences between media were assessed from the estimated marginal means of the model (emmeans package v2.0.4) [22], with p-values adjusted for multiple comparisons (three pairwise tests per metric) using the Bonferroni correction.

Data handling, statistical analyses, and visualisation were conducted using R packages including renv (v1.3.1) [24] for package management, tidyverse (v2.0.0) [25] for data manipulation and plotting, readxl (v1.5.0) [26] for importing Excel files, colorspace (v2.1-3) [27] for colour adjustments, scales (v1.4.0) [28] for axis formatting, patchwork (v1.3.2) [29] for combining plots, ggpubr (v1.0.0) [30] for statistical annotations, rstatix (v1.1.0) [31] for significance-level formatting, nlme (v3.1-170) [21] for generalised least squares models of growth rates, lme4 (v2.0-6) [23] for linear mixed-effects models, emmeans (v2.0.4) [22] for estimated marginal means and contrasts, and SuperPlotR (v0.1.2) [32] for identifying representative technical replicates.

## 3 Results

### 3.1 Effects of carbon source on early-stage biofilm formation

To assess the biofilm-specific growth rate across three carbon sources – glucose, glycerol, and succinate – in M9 medium with and without CAA, wells of a 96-well μ-Plate (ibidi GmbH) were inoculated with a diluted overnight culture, and cells were allowed to propagate on the surface over a period of 8 h. At hourly time points, the biofilms were fixed, rinsed to remove any unattached cells, and stained before confocal laser scanning microscopy (CLSM) imaging, which enabled quantification of biofilm parameters such as cell number and colony size, as described in the Methods section.

At the 0 and 1 h time points, there were typically 1–2 surface-attached *P. fluorescens* cells per field of view (FOV) (106.5 × 106.5 μm, ∼1.13 × 10^4^ μm^2^). All cells in the FOV were counted and considered as one colony. Like the 2 h time point, these time points were not used to calculate μ. At 8 h, the number of biofilm cells per colony (mean ± standard deviation, SD) was 1677 ± 621 in glucose (Fig. 1a), 1323 ± 370 in glycerol (Fig. 1b), and 386 ± 123 in succinate (Fig. 1c). Without CAA, colonies remained much smaller, with an average of 80 cells in glucose, 21 in glycerol, and 54 in succinate at 8 h (Fig. 3d–f).

**Fig. 1:**
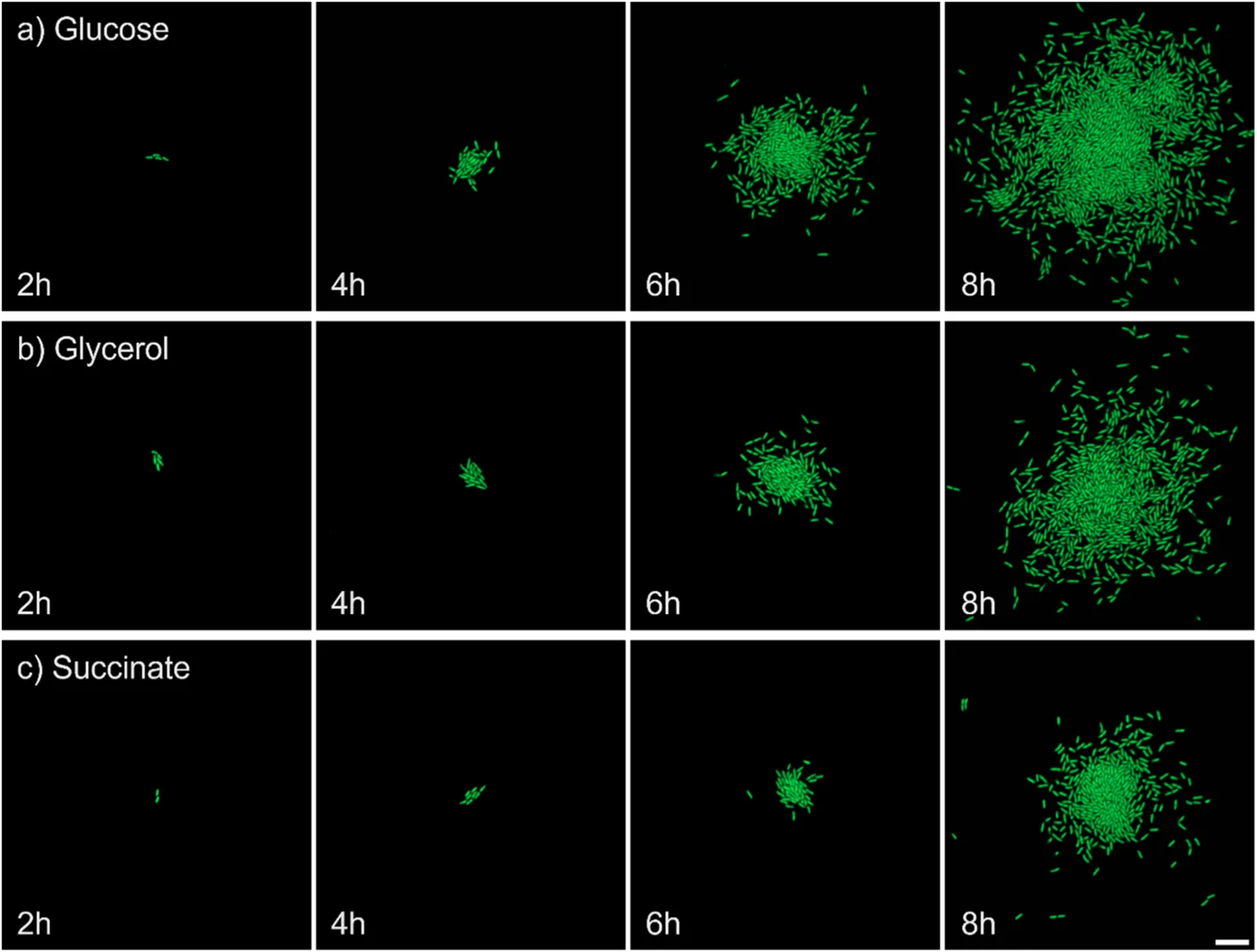
Time progression of *P. fluorescens* WCS365 biofilm development. Representative CLSM images of biofilms stained with SYTO 9 (green) to visualise cells, grown for 2, 4, 6, and 8 h in M9 with CAA and a) glucose, b) glycerol, and c) succinate. Images are representative of 10 colonies each from three independent biological replicates. Scale bar represents 10 μm. (For interpretation of the references to colour in this figure legend, the reader is referred to the Web version of this article.)

In all media conditions, the number of surface-attached cells increased throughout the 8 h assay. With CAA, colonies in glucose and glycerol were of similar size up to 3 h and diverged from 4 h onwards, whereas colonies in succinate were smaller at every time point (Fig. 1, Fig. 3). By 8 h, colonies with CAA contained an average of more than 300 cells across all carbon sources, and biofilm volume was highest in glucose and lowest in succinate (Fig. 2a).

**Fig. 2:**
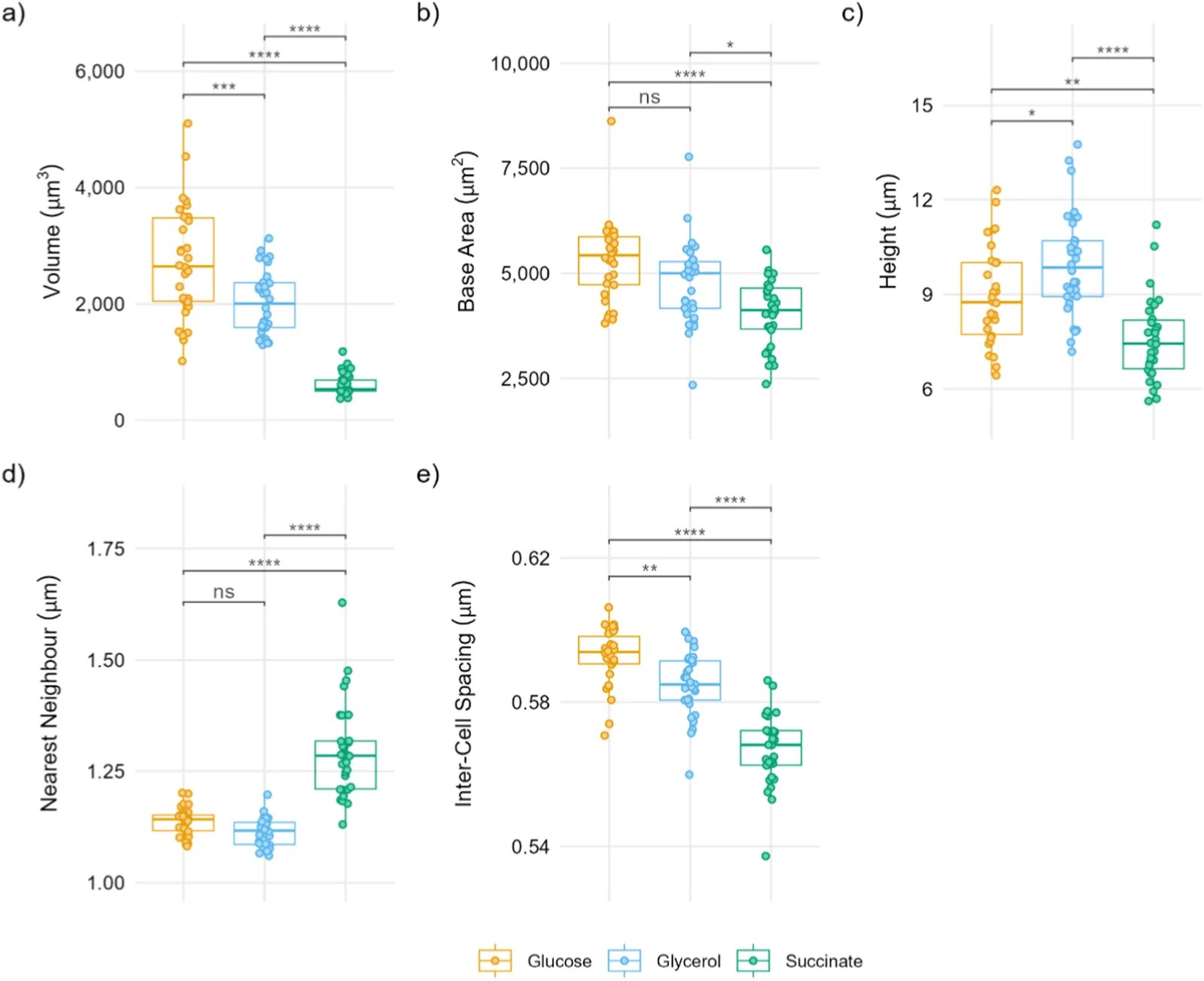
Comparison of biofilm volume, base area, height, and cell-packing architecture of *P. fluorescens* WCS365 across carbon sources at 8 h. Boxplots show a) biofilm volume (μm^3^), b) biofilm base area (μm^2^), c) biofilm height (μm), d) mean distance to nearest neighbour (μm), and e) mean inter-cell spacing (μm) for biofilms grown in M9 with CAA and either glucose, glycerol, or succinate. Experiments were performed with three independent biological replicates, with 10 technical replicate colonies imaged per biological replicate. Boxes represent the interquartile range (IQR), horizontal lines indicate medians, whiskers extend to the most extreme values within 1.5 × IQR of the box, and points show individual colony-level measurements. Pairwise comparisons between carbon sources were performed using linear mixed-effects models (carbon source as a fixed effect, biological replicate as a random intercept, to account for technical replicates nested within biological replicates), with Bonferroni-corrected pairwise contrasts of estimated marginal means. ns indicates not significant, * indicates *p* < 0.05, ** indicates *p* < 0.01, *** indicates *p* < 0.001, and **** indicates *p* < 0.0001.

**Fig. 3:**
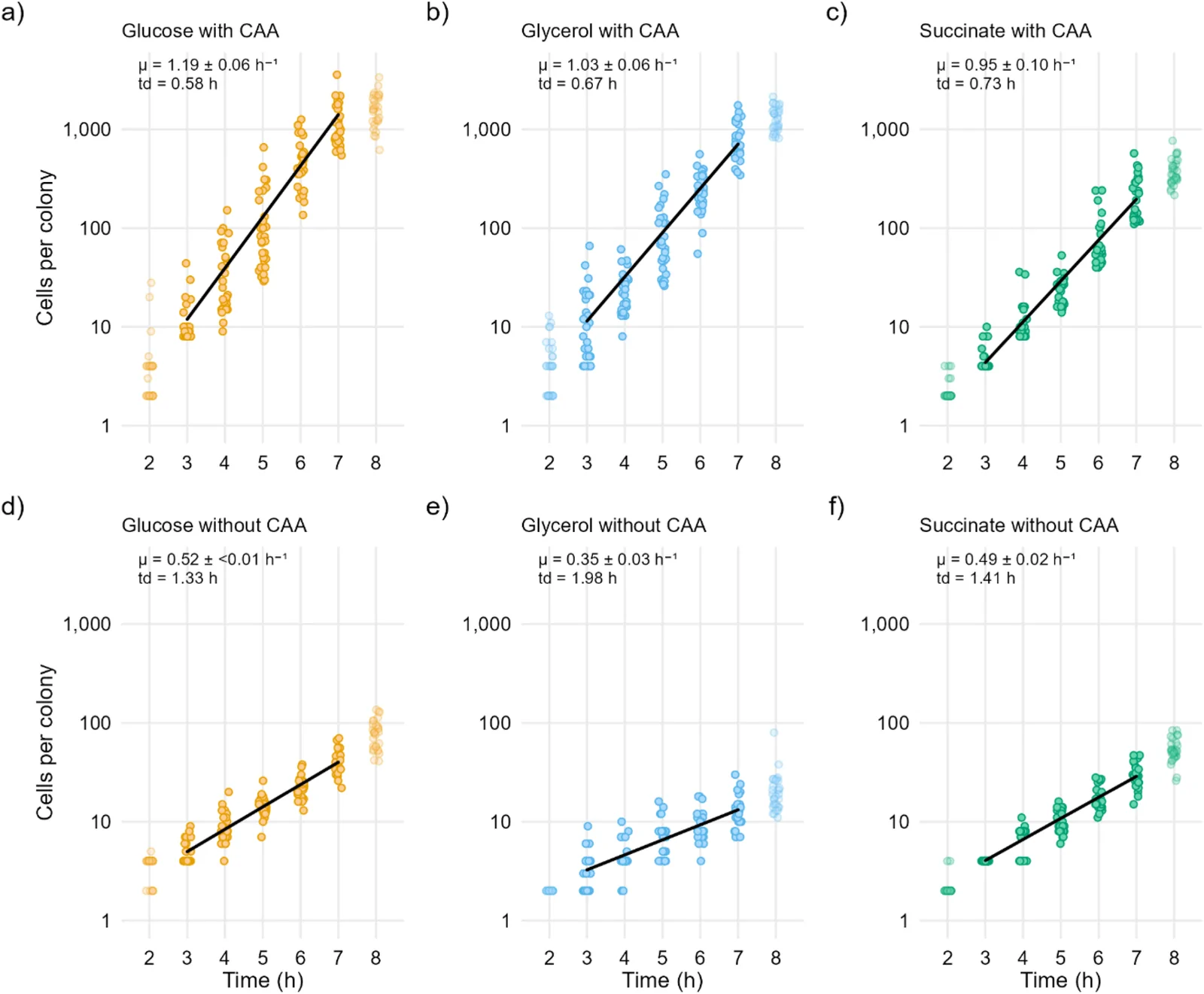
Biofilm growth of *P. fluorescens* WCS365 grown in a) glucose, b) glycerol, and c) succinate with CAA, and d) glucose, e) glycerol, and f) succinate without CAA. Points show the number of segmented cells per colony (10 colonies from each of three independent biological replicates) on a log_10_ scale. Lines show the mean of the log-linear regressions fitted to each biological replicate over the exponential phase (3–7 h); faded points were not used in the fits. The specific growth rate (μ, mean ± SD of three biological replicates) and the doubling time (t_d_) are given in each panel.

Automated segmentation agreed closely with manual annotation (Supplementary Table S2, Figs. S5 and S6). Across 30 validation stacks, precision was 0.92, recall 0.81, and F1 0.86, with similar values for each carbon source, with and without CAA, and at low and high densities. Only 4 of 707 manually marked cells were not detected. Most errors arose from touching cells, mainly daughter cells after division being joined into a single object, resulting in automated counts that were on average 11% lower than manual counts. This undercount was constant over time and therefore altered the specific growth rate by only −0.010 h^−1^ (95% confidence interval, CI, −0.068 to +0.047).

### 3.2 Quantification of biofilm structural properties across carbon sources

At 8 h with CAA, biofilm structural properties differed across carbon sources as assessed by biofilm volume, height, and base area. Biofilm volume, which is the summed volume of all segmented cells within each biofilm, was highest in glucose-grown biofilms (2717 ± 976 μm^3^; mean ± SD), intermediate in glycerol (2028 ± 554 μm^3^), and lowest in succinate (617 ± 188 μm^3^; Fig. 2a). Glucose biofilms had significantly greater volumes than glycerol (*p* = 0.0002) and succinate (*p* < 0.0001), and glycerol biofilms had significantly greater volumes than succinate (*p* < 0.0001).

Biofilm base area, quantified as the area of a fitted ellipse describing the biofilm footprint at the surface, followed a similar but less separated trend, with glucose exhibiting the largest base areas (5307 ± 967 μm^2^), glycerol intermediate (4796 ± 993 μm^2^), and succinate the smallest (4084 ± 825 μm^2^; Fig. 2b). Succinate biofilms had significantly smaller base areas than both glucose (*p* < 0.0001) and glycerol (*p* = 0.012), while glucose and glycerol did not differ significantly (*p* = 0.11).

Biofilm height, defined as the global range spanning the 1st to 99.5th percentile of z-coordinates across all segmented cells within a colony, also differed across carbon sources at 8 h, though the order did not match that observed for volume and base area. Glycerol-grown biofilms were tallest (9.9 ± 1.7 μm), glucose biofilms were intermediate (8.9 ± 1.6 μm), and succinate biofilms were shortest (7.6 ± 1.3 μm; Fig. 2c). Glycerol biofilms were significantly taller than both glucose (*p* = 0.036) and succinate (*p* < 0.0001), and glucose biofilms were significantly taller than succinate (*p* = 0.002). Across all three metrics, succinate biofilms were consistently the smallest. Glucose and glycerol biofilms occupied similar base areas, but glucose biofilms reached significantly greater volume despite being shorter than glycerol, indicating that glucose biofilms contained more cells within a similar footprint rather than growing taller.

Biofilm architecture also varied across carbon sources when assessed by cell-packing metrics, though not always in line with the volume and base area order described above. Distance to nearest-neighbour, the mean centroid-to-centroid distance between each cell and its closest neighbour, was greatest in succinate biofilms (1.29 ± 0.11 μm) compared with glucose (1.14 ± 0.03 μm) and glycerol (1.11 ± 0.03 μm) biofilms (Fig. 2d). Succinate biofilms had significantly greater nearest-neighbour distances than both glucose (*p* < 0.0001) and glycerol (*p* < 0.0001), while glucose and glycerol did not differ significantly (*p* = 0.51).

In contrast, inter-cell spacing, the mean boundary-to-boundary distance between each cell and neighbouring cells within a 20-voxel screening range, was lowest in succinate biofilms (0.567 ± 0.010 μm) compared with glucose (0.593 ± 0.008 μm) and glycerol (0.585 ± 0.009 μm) biofilms (Fig. 2e). Succinate biofilms had significantly narrower inter-cell spacing than both glucose (*p* < 0.0001) and glycerol (*p* < 0.0001), and glycerol biofilms had slightly but significantly narrower spacing than glucose (*p* = 0.003). This trend is the reverse of that observed for nearest-neighbour distance. Inter-cell spacing only includes gaps up to the 20-voxel screening range (∼1.8 μm) and excludes touching cells, so it reflects the spacing between closely neighbouring cells, whereas nearest-neighbour distance also captures the wider spacing between cells in the sparser succinate biofilms.

### 3.3 Quantitative analysis of biofilm growth kinetics

To visualise the early-stage biofilm growth kinetics, log-transformed cell counts were plotted against time for *P. fluorescens* grown in M9 with glucose, glycerol, or succinate, with and without CAA (Fig. 3). With CAA, glucose-grown biofilms had the fastest specific growth rate (μ = 1.193 ± 0.056 h^−1^), glycerol-grown biofilms were intermediate (μ = 1.032 ± 0.064 h^−1^), and succinate-grown biofilms were slowest (μ = 0.953 ± 0.096 h^−1^; Table 1), reflected in the steeper slope of the glucose growth curve compared with glycerol and succinate (Fig. 3a–c).

**Table 1:** Summary of biofilm growth kinetics of *P. fluorescens* WCS365 in M9 with glucose, glycerol, or succinate, with and without CAA. Specific growth rate (μ, mean ± SD) and coefficient of determination (R^2^, mean) were obtained by log-linear regression of cell number against time over 3–7 h for each of three independent biological replicates, using the mean of 10 colonies per replicate. Doubling time (t_d_) was calculated as ln(2)/μ from the mean μ.

Medium	μ (h-1, mean ± SD)	R^2^ (mean)	t_d_ (h)
Glucose + CAA	1.193 ± 0.056	0.988	0.581
Glycerol + CAA	1.032 ± 0.064	0.971	0.672
Succinate + CAA	0.953 ± 0.096	0.981	0.727
Glucose – CAA	0.521 ± 0.001	0.995	1.330
Glycerol – CAA	0.350 ± 0.028	0.975	1.982
Succinate − CAA	0.490 ± 0.025	0.984	1.414

Correspondingly, t_d_ increased from glucose to succinate, with the shortest t_d_ observed in glucose (0.581 h), an intermediate value in glycerol (0.672 h), and the longest t_d_ in succinate (0.727 h). Exponential regression fits showed high coefficients of determination across all six conditions (mean R^2^ = 0.971–0.995), indicating that the exponential growth model adequately described early-stage biofilm growth for each carbon source. With CAA, early-stage biofilm specific growth rates therefore declined from glucose to glycerol to succinate.

Without CAA, biofilms grew more slowly in all three carbon sources (Fig. 3d–f, Table 1). Glucose biofilms again grew fastest (μ = 0.521 ± 0.001 h^−1^, t_d_ = 1.330 h), but succinate biofilms (μ = 0.490 ± 0.025 h^−1^, t_d_ = 1.414 h) grew faster than glycerol biofilms (μ = 0.350 ± 0.028 h^−1^, t_d_ = 1.982 h), such that the order of glycerol and succinate was reversed compared with media containing CAA.

### 3.4 Planktonic growth kinetics under equivalent nutrient conditions

For comparison with biofilm growth, planktonic growth experiments were performed in shake flasks containing M9 medium with each respective carbon source (glucose, glycerol, or succinate) with and without CAA. OD_600_ was measured every 60 min over 8 h. Specific growth rates (μ) were calculated by linear regression of the natural logarithm of dilution-corrected OD_600_ against time over the exponential phase (2–5 h for glucose and glycerol, and 2–6 h for succinate with CAA; 3–7 h for glucose and succinate without CAA), and planktonic growth was visualised by plotting log-transformed dilution-corrected OD_600_ against time for *P. fluorescens* (Fig. 4). The experiment was performed with three independent biological replicates, each with three technical replicates.

**Fig. 4:**
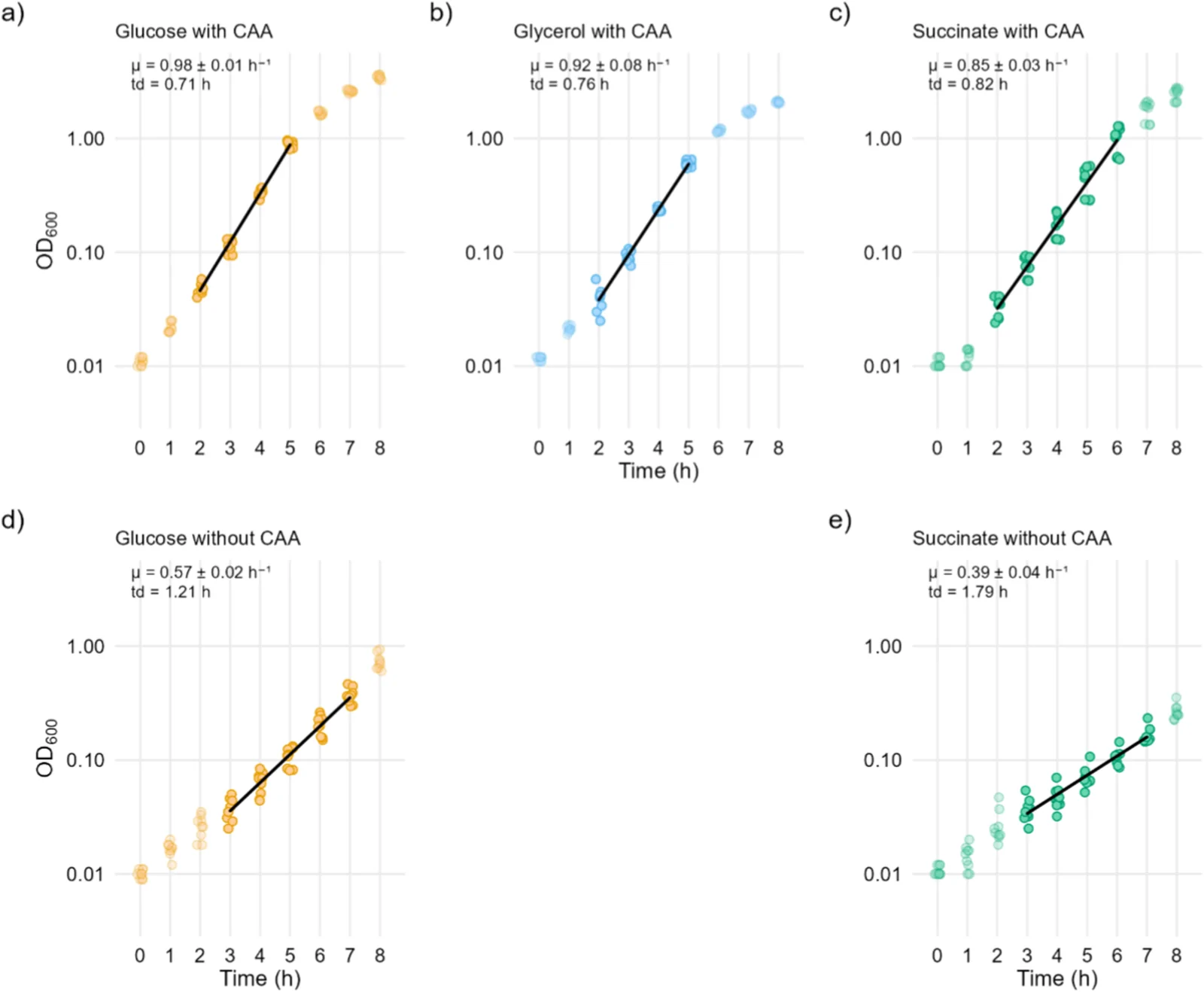
Planktonic growth of *P. fluorescens* WCS365 in a) glucose, b) glycerol, and c) succinate with CAA, and d) glucose and e) succinate without CAA. Points show the dilution-corrected optical density (OD_600_) of three technical replicates each from three independent biological replicates, plotted on a log_10_ scale. Lines show the mean of the log-linear regressions fitted to each biological replicate over the exponential phase (2–5 h for glucose and glycerol, and 2–6 h for succinate with CAA; 3–7 h for glucose and succinate without CAA); faded points were not used in the fits. The specific growth rate (μ, mean ± SD of three biological replicates) and the doubling time (t_d_) are given in each panel. Glycerol without CAA is not shown because no increase in OD_600_ was detected within the 8 h assay.

With CAA, planktonic μ followed the same order of carbon sources as biofilm μ. Cells grown in glucose showed the highest mean μ (0.982 ± 0.010 h^−1^), followed by glycerol (0.915 ± 0.076 h^−1^), and succinate (0.849 ± 0.029 h^−1^; Table 2). Accordingly, t_d_ was shortest in glucose (0.706 h), intermediate in glycerol (0.757 h), and longest in succinate (0.817 h). Linear regressions of log-transformed OD_600_ values yielded high coefficients of determination across all conditions (R^2^ = 0.991–0.999), indicating that exponential growth was well described by the fitted models. Without CAA, planktonic cultures grew more slowly, with μ = 0.571 ± 0.023 h^−1^ (t_d_ = 1.215 h) in glucose and 0.386 ± 0.041 h^−1^ (t_d_ = 1.795 h) in succinate (Fig. 4d and e, Table 2). In glycerol without CAA, no increase in OD_600_ was detected within the 8 h assay.

**Table 2:** Summary of planktonic growth kinetics of *P. fluorescens* WCS365 in M9 with glucose, glycerol, or succinate, with and without CAA. Specific growth rate (μ, mean ± SD) and coefficient of determination (R^2^, mean) were obtained by log-linear regression of dilution-corrected OD_600_ against time over the exponential phase defined for each condition across three independent biological replicates, using the mean of three technical replicates per replicate. Doubling time (t_d_) was calculated as ln(2)/μ from the mean μ. No increase in OD_600_ was detected in glycerol without CAA within the 8 h assay.

Medium	Window	μ (h^−1^, mean ± SD)	R^2^ (mean)	t_d_ (h)
Glucose + CAA	2–5 h	0.982 ± 0.010	0.998	0.706
Glycerol + CAA	2–5 h	0.915 ± 0.076	0.997	0.757
Succinate + CAA	2–6 h	0.849 ± 0.029	0.999	0.817
Glucose − CAA	3–7 h	0.571 ± 0.023	0.997	1.215
Succinate − CAA	3–7 h	0.386 ± 0.041	0.991	1.795

In dilution series, OD_600_ was slightly less than directly proportional to cell density, with a log-log slope of 0.94 (0.92–0.96 across carbon sources; a slope of 1 indicates proportionality), such that readings of 0.4 were 15–21% lower than expected from the most dilute samples in glucose and glycerol, and readings of 0.3 were 4–11% lower in succinate (Supplementary Fig. S7). In growing cultures, however, CFU per OD_600_ unit did not change between 2 and 6 h (0.05% per hour, p = 0.98), and specific growth rates from CFU and OD_600_ did not differ (mean difference −0.03 h^−1^, 95% CI −0.11 to +0.05; Supplementary Fig. S8). OD_600_ therefore tracked viable cell number over the window used to estimate planktonic growth rates with CAA. In biofilms with CAA, the mean volume per segmented cell was 1.4–1.6 μm^3^ between 3 and 7 h and differed by less than 3% between carbon sources.

### 3.5 Comparison of biofilm and planktonic growth rates

With CAA, biofilm specific growth rates were on average 15% higher than planktonic growth rates (95% CI 7–24%, *p* = 0.002), and this difference did not depend significantly on carbon source (lifestyle × carbon source interaction, *p* = 0.42; Table 1, Table 2, Supplementary Table S3). For individual carbon sources, biofilm growth rates were 21% (glucose), 13% (glycerol), and 12% (succinate) higher, but none of these differences remained significant after correction for multiple comparisons. Growth rates differed between carbon sources (*p* = 0.015), with the same order in both lifestyles (glucose > glycerol > succinate), although no individual pair of carbon sources differed significantly after correction (Supplementary Table S3).

The comparison depended on the fitting window. With CAA, biofilm growth rates over windows of 2–6, 2–7, and 2–8 h differed by at most 10% from those over 3–7 h, whereas planktonic growth rates decreased by up to 25% when the window extended beyond the exponential phase (Supplementary Table S1). When the same window was applied to both lifestyles, the biofilm advantage in glucose and glycerol therefore increased to 30–51% for common windows ending at 7 or 8 h. Because these common windows include decelerating planktonic growth (Supplementary Fig. S4), growth rates were compared over the exponential phase of each lifestyle.

Removing CAA while keeping total carbon constant reduced specific growth rates in every condition (Table 1, Table 2). Biofilm growth rates decreased by 56% in glucose, 66% in glycerol, and 49% in succinate, and planktonic growth rates by 42% in glucose and 54% in succinate. In glycerol without CAA, no increase in OD_600_ was detected within the 8 h assay, whereas attached cells grew at 0.350 h^−1^. Without CAA, the biofilm growth rate was 9% lower than the planktonic growth rate in glucose and 27% higher in succinate, and neither difference was significant (adjusted *p* = 0.30 and 0.21). Removing CAA reduced the ratio of biofilm to planktonic growth rate in glucose by 25% (95% CI 14–34%, adjusted *p* = 0.003), whereas the change in succinate (+14%) was not significant (adjusted *p* = 0.43; Supplementary Table S3).

## 4 Discussion

### 4.1 Comparative growth dynamics of planktonic and biofilm populations

With CAA, surface-attached *P. fluorescens* grew on average 15% faster than planktonic cells (95% CI 7–24%), and this difference did not depend significantly on the carbon source. The larger and substrate-dependent differences suggested by fitting both lifestyles over a fixed 2–8 h window arose because that window included the deceleration of planktonic cultures after 5–6 h, whereas attached cells continued to grow exponentially until 7 h (Supplementary Table S1). The two lifestyles were measured with different methods: cell counts for biofilms and OD_600_ for planktonic cultures. In growing planktonic cultures, however, OD_600_ tracked the number of CFU over the fitted window (Supplementary Fig. S8), so the faster growth of attached cells is unlikely to reflect the use of OD_600_ as a proxy for cell number, although differences in planktonic cell size or aggregation cannot be excluded.

Earlier studies concluded that attachment confers no inherent, consistent metabolic advantage, and that differences in activity between attached and free-living cells depend on the substratum, the substrate supplied, and changes in the local environment rather than on adhesion itself [4], [33], [34], [35]. Even the 2- to 5-fold higher glucose assimilation of attached *P. fluorescens* reported by Fletcher [36] was later attributed to substrate depletion among the free-living cells [4]. In the present study, planktonic growth rates were estimated only over the exponential phase, so substrate depletion is unlikely to explain the modest advantage of attached cells. Possible explanations include the much higher local cell density within a biofilm, where metabolites released by neighbouring cells could be shared, and surface sensing through c-di-GMP signalling [37], but neither was tested here.

### 4.2 Influence of carbon source and casamino acids on growth rate

With CAA, growth rates followed the same order in both lifestyles, glucose > glycerol > succinate. This order is consistent with how close to hexose phosphates each substrate enters central metabolism. In the related species *P. putida*, glucose is converted mainly to 6-phosphogluconate and catabolised through the Entner-Doudoroff pathway [38], [39]. Glycerol enters at the triose-phosphate level and elicits a gluconeogenic response to regenerate hexose phosphates [40]. Succinate enters the tricarboxylic acid cycle, such that hexose-phosphate precursors must be regenerated through the full gluconeogenic pathway. This interpretation was not tested directly.

Despite matched total carbon, growth rates were 42–66% lower without CAA in all conditions where growth was detected. CAA therefore acted mainly as a nutritional supplement, most plausibly by supplying preformed amino acids that cells would otherwise synthesise from the primary carbon source. Growth in CAA-supplemented medium reflects the combined effects of the primary carbon source and CAA. In CAA-free medium, attached cells grew faster in succinate than in glycerol (0.490 vs 0.350 h^−1^; Table 1), so the order discussed above held only when CAA was present. If CAA had mainly repressed catabolism of the primary carbon source [41], its removal should have increased growth in glucose and glycerol, which was not observed. Without CAA, the biofilm advantage in glucose was lost, whereas in glycerol, attached cells grew while no increase in OD_600_ was detected within the 8 h assay, indicating that the relationship between the two lifestyles depends on the nutritional context.

### 4.3 Influence of carbon source on biofilm structural properties and the role of the extracellular matrix

At 8 h, biofilm structure differed between carbon sources (Fig. 2). Biofilm volume and base area followed the order of the growth rates, with glucose biofilms 4.4-fold larger in volume than succinate biofilms and 34% larger than glycerol biofilms, consistent with faster growth producing more cells over the same period. Other metrics did not follow this order. Glycerol biofilms were the tallest, 11% taller than glucose biofilms and 31% taller than succinate biofilms. Succinate biofilms had a 14–16% larger mean distance to the nearest neighbouring cell, suggesting that cells were spread more sparsely across the surface but packed at least as closely where they occurred together, as indicated by their smaller inter-cell spacing (Fig. 2e).

Part of these differences may reflect the extracellular matrix rather than the cells themselves. The matrix of bacterial biofilms consists mainly of polysaccharides, proteins, nucleic acids, and lipids, and holds cells together and to the surface [42]. In *P. fluorescens* WCS365, irreversible attachment requires the large surface adhesin LapA [43], and in *P. fluorescens* Pf0-1 its localisation at the cell surface is controlled by phosphate availability through c-di-GMP signalling [6]. The carbon source can also influence the amount of exopolysaccharide produced by *Pseudomonas* species [44], and glycerol promotes surface attachment and alters colony morphology in *P. aeruginosa* [45]. Because the matrix occupies space between cells and mediates cell–cell interactions, it can determine how closely cells pack and how biofilms grow in height, and in *Vibrio cholerae* the local order and overall architecture of biofilms arise from such matrix-dependent cell–cell interactions [46]. Carbon-source-dependent differences in matrix production or composition could therefore contribute to the greater height of glycerol biofilms and the more dispersed arrangement of succinate biofilms, independently of growth rate. All architecture metrics used here were calculated from segmented cells and do not include the matrix, which was not stained in this study, so this possibility remains to be tested.

### 4.4 Broader ecological and applied implications

In natural environments such as the rhizosphere, the nutrients available to bacteria change over time, for example as the composition of root exudates shifts with plant development [47]. The present results suggest that the relative growth of attached and free-living cells in such environments is not fixed but depends on which nutrients are available, and that the carbon source shapes biofilm structure in ways not explained by growth rate alone. Managing the nutrient composition of a system may therefore complement other strategies for controlling biofilm growth and structure in settings where biofilms are undesirable, such as in industrial biofouling, or beneficial, such as in wastewater treatment.

### 4.5 Limitations

Despite these insights, several limitations of the present study should be acknowledged. First, all experiments were conducted using a single bacterial species and strain, *P. fluorescens* WCS365, and the extent to which the results generalise to other taxa remains uncertain. Second, the study focused on early-stage biofilms under static conditions and did not address later stages of biofilm maturation, where structural heterogeneity and nutrient limitation may alter growth dynamics. Third, planktonic and biofilm growth rates were measured with different methods. OD_600_ tracked CFU over the fitted window, but planktonic cell size, morphology, and aggregation were not measured directly. Fourth, populations were sampled hourly, so the exponential phase of planktonic cultures with CAA contained only four or five time points, and the comparison between lifestyles depended on the fitting window (Supplementary Table S1). Fifth, amino acid uptake, catabolite repression, and biosynthetic pathway activity were not measured, so the mechanisms behind the effects of CAA remain partially unresolved. Sixth, the EPS was not stained or quantified, so its contribution to the differences in biofilm structure could not be assessed. Finally, with three biological replicates per group, the study had limited power to resolve differences for individual carbon sources, which are therefore reported as effect sizes with confidence intervals.

These limitations point to several directions for future research. Direct measurement of substrate and amino acid uptake and biomass yield (Y_X/S_) in both lifestyles would show how CAA contributes to growth, and reporter or transcriptomic measurements of genes regulated by the catabolite repression proteins Crc and Hfq [41], [48] would test whether catabolite repression contributes to the differences between carbon sources. Imaging planktonic cells with the same segmentation pipeline would enable direct comparison of cell size between lifestyles and allow growth rates to be calculated from total biovolume as well as cell counts, reducing the uncertainty inherent in comparing OD-based and count-based growth rates. Staining EPS and adhesins alongside the cells, and quantifying matrix production in each carbon source, would test whether the matrix explains the differences in biofilm height and cell packing that were not explained by growth rate. Extending this approach to flow conditions, a range of substrate concentrations, and other *P. fluorescens* strains would clarify the generality of the present findings.

## 5 Concluding remarks

This study compared the growth kinetics of surface-attached and planktonic *P. fluorescens* WCS365 in glucose, glycerol, and succinate, with and without CAA, using single-cell confocal imaging to count cells within early biofilms. With CAA, attached cells grew on average 15% faster than planktonic cells, and this difference did not depend significantly on the carbon source. Removing CAA slowed growth in both lifestyles and removed the biofilm advantage in glucose, indicating that the relationship between the two lifestyles depends on the nutritional context rather than attachment alone.

The carbon source also shaped biofilm structure. Biofilm volume and base area followed the order of the growth rates (glucose > glycerol > succinate), consistent with faster growth producing more cells, whereas biofilm height and nearest-neighbour distance did not, suggesting that factors other than growth rate, such as the EPS, contribute to the spatial organisation of early *P. fluorescens* biofilms.

## CRediT authorship contribution statement

**Jacobus Brink:** Writing – original draft, Visualization, Validation, Software, Methodology, Investigation, Formal analysis, Data curation, Conceptualization. **Dishon Wayne Hiebner:** Writing – review & editing, Supervision, Software, Methodology, Investigation. **Eoin Casey:** Writing – review & editing, Supervision, Resources, Project administration, Funding acquisition, Conceptualization.

## Code availability

The segmentation code is publicly available on GitHub at https://github.com/JacobusBrink/stardist_batch_processing_biofilms. The R code used for growth kinetics, statistical analysis, and data visualisation is available at https://github.com/JacobusBrink/pf_biofilm_growth_advantage.

## Declaration of generative AI use

Generative artificial intelligence (AI) tools were used in the preparation of this manuscript. Specifically, Claude (Anthropic, Claude Opus 5.5, accessed *via* Claude Code) was used for coding assistance, including development of R scripts for data visualisation and statistical analysis, as well as language-related assistance, including paraphrasing and editing of text. The tool was employed to improve clarity, structure, and readability of the manuscript without generating new scientific content, data, or interpretations. All AI-assisted outputs were carefully reviewed, edited, and validated by the authors, who take full responsibility for the accuracy, originality, and integrity of the final manuscript.

## Declaration of competing interest

The authors declare the following financial interests/personal relationships which may be considered as potential competing interests:Eoin Casey reports financial support was provided by European Research Council. If there are other authors, they declare that they have no known competing financial interests or personal relationships that could have appeared to influence the work reported in this paper.

## Data Availability

The datasets generated and analysed during this study are available in the Zenodo repository, https://doi.org/10.5281/zenodo.17977574.
