# Supplementary material for: Single-cell counting reveals a modest, nutrient-dependent growth advantage of early Pseudomonas fluorescens biofilms over planktonic cells
Source: Biofilm. 2026 Oct 29;12:100408. doi: 10.1016/j.bioflm.2026.100408 (PMC13769525; doi:10.1016/j.bioflm.2026.100408)
Supplement: Supplementary file 1 [file mmc1.docx]

Single-cell counting reveals a modest, nutrient-dependent growth advantage of early *Pseudomonas fluorescens* biofilms over planktonic cells

# 1. Supplementary Information

## 1.1 Segmentation Validation

Segmentation was validated against manual annotation on 30 image stacks of the re-segmented dataset. Stacks were selected at random, using a reproducible script, before any annotation was done. With CAA, the set covered each carbon source (glucose, glycerol, and succinate) at every hour from 2 to 8 h. Without CAA, it covered each carbon source at 2, 5, and 8 h. Biological replicates were rotated across time points, and technical replicates were chosen at random. Stacks with 50 or fewer segmented objects were annotated whole (low density). Denser stacks were cropped to a 9-slice band (about 2.8 µm) around the densest layer, with an *xy* window containing about 30 cells (high density). This is where the merging of touching cells is most likely.

One annotator marked every cell in Fiji (Schindelin et al., 2012) on the raw images, with one click per cell on the slice where it appeared sharpest. Images were given random codes, and the segmentation was not visible during annotation. A click was matched to the segmented object containing it, or to the nearest object within 3 pixels (0.27 µm) in *xy* and one slice (0.31 µm) in *z*. An object matched by one click was a true positive. An object matched by two or more clicks counted as one true positive, and each extra click as a false negative (merged cells). A click without an object was a false negative, and an object without a click was a false positive. In cropped stacks, objects with less than half of their volume inside the crop were excluded, together with their clicks. Precision, recall, F1 score, and the difference between automated and manual counts were calculated.

To test whether segmentation errors bias the specific growth rate, ln(automated/manual count) was regressed against time for the 15 stacks with CAA in the 3–7 h fitting window. The model used a common slope and a separate intercept for each carbon source. The slope is the error that segmentation adds to µ (h^-1^).

To test cell shape, the same annotator outlined all in-focus cells on one slice of an 8 h biofilm for each carbon source (77 cells). Manual outlines were matched one-to-one to segmented cell cross-sections by maximising the Intersection over Union (IoU), and outlines touching the image edge were excluded.

At 8 h, 50 of the 77 outlined cells (65%) matched a segmented cell with an IoU of at least 0.5, with a mean IoU of 0.68 for these cells. Matched cells agreed in size (median automated/manual area ratio 1.04) and position (median centroid offset 0.11 µm). In the cropped stacks, the mean nearest-neighbour distance between cells was 1.06 µm from the manual clicks and 1.08 µm from the segmented cells.

## 1.2 Supplementary Tables

### 1.2.1 Table S1

Supplementary Table S1. Sensitivity of specific growth rates and of the biofilm versus planktonic comparison to the fitting window

Growth rates are mean ± SD of three biological replicates; biofilm growth rates from segmented cell counts and planktonic growth rates from OD_600_ multiplied by the dilution factor. Selected windows are those used in the main text; the other rows apply the same window to both lifestyles. Differences are from a generalised least squares model of the log-transformed growth rates with a separate variance for each group, fitted separately for each scenario; 95% CI and *p*-values are Bonferroni-adjusted for the five biofilm versus planktonic comparisons within each scenario. No planktonic data were recorded for glycerol without CAA.

### 1.2.2 Table S2

Supplementary Table S2. Segmentation accuracy against manual annotation

Count error = (automated − manual) / manual × 100, pooled over the images in each group.

### 1.2.3 Table S3

Supplementary Table S3. Statistical analysis of specific growth rates

Replicate-level growth rates (*n* = 3 biological replicates per group) were log-transformed and analysed with generalised least squares models allowing a separate variance for each group, with Satterthwaite degrees of freedom. A and B: joint *F* tests. Model B is restricted to glucose and succinate because no planktonic data were recorded for glycerol without CAA. C–E: percentage differences in growth rate with 95% CI. The average over carbon sources in C is unadjusted; the individual comparisons are Bonferroni-adjusted within each family (C and C2 together: five comparisons; D: two; E: six). In D, values are the change in the biofilm/planktonic ratio when CAA was removed.

## 1.3 Supplementary Figures

### 1.3.1 Figure S1

Supplementary Figure S1. Planktonic growth curves of *P. fluorescens* WCS365 for each biological replicate (columns) and condition (rows). Points show the mean dilution-corrected OD_600_ of three technical replicates ± SD, plotted on a log_10_ scale. Filled points were used to estimate the specific growth rate (µ) and open points were not. The line shows the log-linear regression over the fitting window, and the window, µ, and R^2^ are given in each panel. Glycerol without CAA is not shown because no increase in OD_600_ was detected within the 8 h assay.

### 1.3.2 Figure S2

Supplementary Figure S2. Biofilm growth curves of *P. fluorescens* WCS365 for each biological replicate (columns) and condition (rows). Points show the mean number of segmented cells per colony ± SD of 10 colonies, plotted on a log_10_ scale. Lower error bars that would fall below one-tenth of the mean, including zero or below, are cut off at one tenth of the mean so that they remain visible on the log scale. Filled points
(3–7 h) were used to estimate the specific growth rate (µ) and open points were not. The line shows the log-linear regression over this window, and the window, µ, and R^2^ are given in each panel.

### 1.3.3 Figure S3

Supplementary Figure S3. Hour-to-hour specific growth rate, ln(y_t_/y_t−1_), of planktonic (OD_600_) and biofilm (cells per colony) populations with (top) and without (bottom) CAA, plotted at the midpoint of each interval. Thin lines show individual biological replicates and thick lines show their mean. Grey shading marks the window used to estimate the specific growth rate. With CAA, the planktonic rate fell after 5 h in glucose and glycerol, and after 6 h in succinate, and all biofilm rates fell between 7 and 8 h. Without CAA, the planktonic rate increased throughout the assay. No planktonic data were recorded for glycerol without CAA.

### 1.3.4 Figure S4

Supplementary Figure S4. Residuals (observed minus fitted ln(*y*)) of the log-linear regressions used to estimate the specific growth rate, for biofilm (top) and planktonic (bottom) populations. Upper rows show the fitting windows used in this study (biofilm 3–7 h; planktonic 2–5 h for glucose and glycerol, and 2–6 h for succinate with CAA, and 3–7 h without CAA) and lower rows show the original 2–8 h window. Points show the three biological replicates (symbols) and lines show their mean. An arch (positive in the middle, negative at the ends) indicates that growth slowed within the window, and a U-shape that growth accelerated.

### 1.3.5 Figure S5

Supplementary Figure S5. Representative segmentation overlays. Raw SYTO 9 images with StarDist-segmented cell outlines (cyan) on the densest slice of 3 h and
7 h biofilms grown with CAA, for glucose, glycerol, and succinate. The 3 h glucose and succinate panels show the whole analysed region; the other panels show the manually annotated region of the colony. Panels are scaled to the same size. Scale bars, 2 µm.

### 1.3.6 Figure S6

Supplementary Figure S6. Segmentation accuracy across carbon sources and timepoints. a) Automated versus manual cell counts per image, plotted on log_10_ scales; the line shows 1:1 agreement. b) Count error ((automated − manual)/manual × 100) and c) recall (fraction of manually identified cells detected by segmentation) per image over time; lines connect images with CAA, and the grey band marks the 3–7 h fitting window. Filled circles, with CAA; open circles, without CAA.

### 1.3.7 Figure S7

Supplementary Figure S7. Linearity of OD_600_ measurements. a) OD_600_ of two-fold serial dilutions of one culture per carbon source with CAA (three independent dilution series; points are the mean of three readings), plotted on log scales. Dashed lines show proportionality extrapolated from the three most dilute points, and solid lines show the calibration curve (quadratic fit in log-log space). b) Deviation of each reading from proportionality; grey shading marks the OD_600_ range used for growth rate estimation.

### 1.3.8 Figure S8

Supplementary Figure S8. OD_600_ and colony-forming units (CFU) in growing planktonic cultures with CAA. a) CFU and OD_600_ of three biological replicate cultures per carbon source, each expressed relative to its 2 h value and plotted on a log_10_ scale. b) Specific growth rates from CFU against those from OD_600_ for each culture, over the fitting window (glucose and glycerol: 2–5 h, succinate: 2–6 h); error bars show the standard error of the regression slope, and the dashed line marks equality.
